# Supplementary material for: Deciphering the Role of ADAMTS6 in the Epithelial–Mesenchymal Transition of Lung Adenocarcinoma Cells
Source: Int J Mol Sci. 2025 Dec 8;26(24):11850. doi: 10.3390/ijms262411850 (PMC12732811; doi:10.3390/ijms262411850)
Supplement: Supplementary file 1 [file ijms-26-11850-s001.zip › Suplementary materials_FINAL.pdf]

Article

Deciphering the Role of ADAMTS6 in the Epithelial–Mesenchymal Transition of Lung Adenocarcinoma Cells

Kirill V. Odarenko, Anastasiya M. Matveeva, Grigory A. Stepanov, Marina A. Zenkova and Andrey V. Markov *

Institute of Chemical Biology and Fundamental Medicine, Siberian Branch of the Russian Academy of Sciences, 630090 Novosibirsk, Russia; k.odarenko@yandex.ru (K.V.O.); anastasiya.maatveeva@gmail.com (A. M. M.); stepanovga@1bio.ru (G. A. S.); marzen@1bio.ru (M.A.Z.)

* Correspondence: andmrkv@gmail.com; Tel.: +7-383-363-51-61

Supplementary Materials

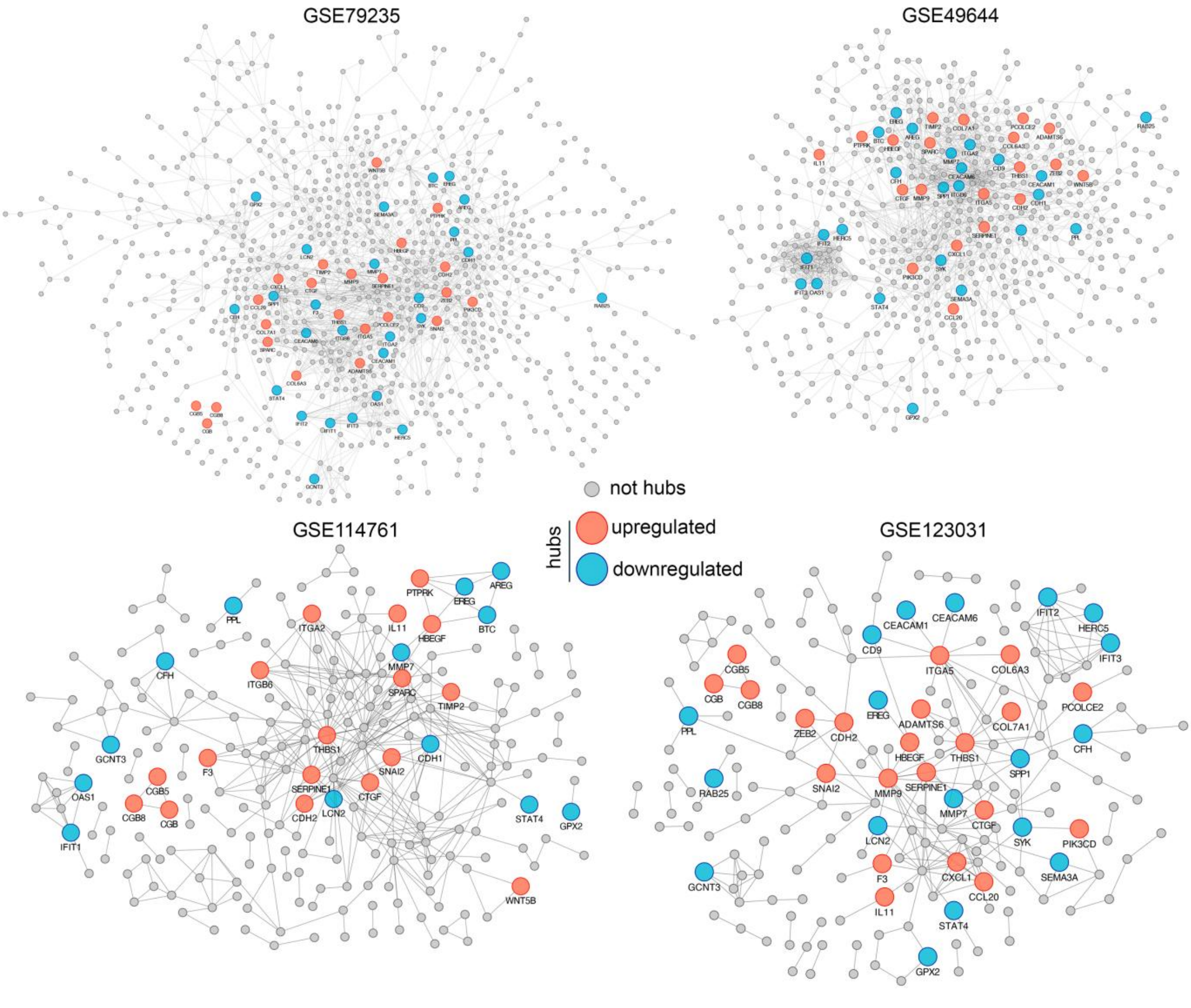

Figure S1. EMT gene networks. Four gene regulatory networks were constructed from differentially expressed gene (DEG) lists derived from four microarray datasets of TGF-β1-induced LUAD cells using the STRING database and visualized in Cytoscape. Hub genes were identified within each network using the CytoHubba plugin, which evaluates 12 topo-

logical criteria. These hubs are represented as large circles, with colors indicating the direction of differential expression changes (e.g., upregulated in red, downregulated in blue).

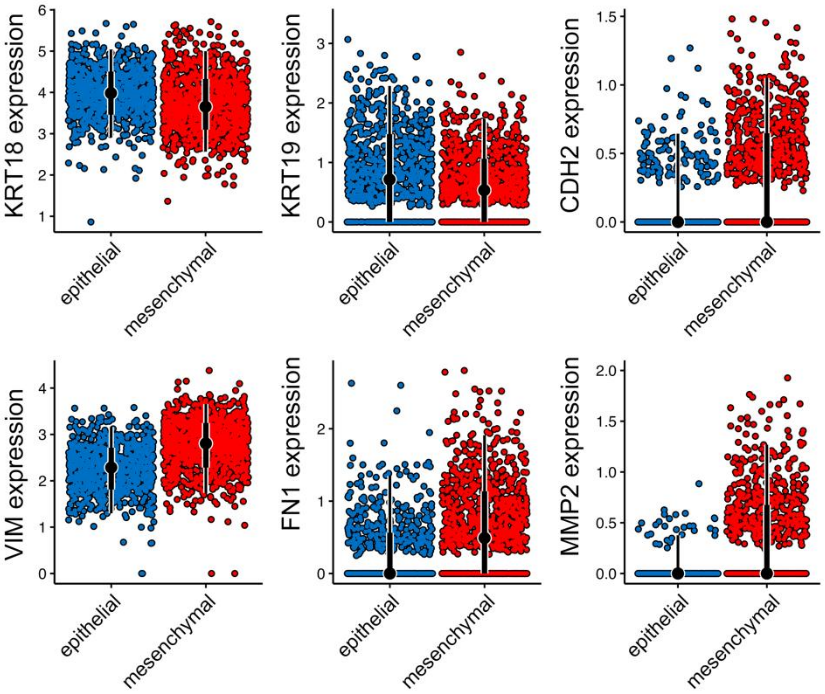

Figure S2. Normalized expression of EMT cell markers in epithelial-like and mesenchymal-like clusters of A549 cells, identified through scRNA-seq analysis of the GSE147405 dataset.

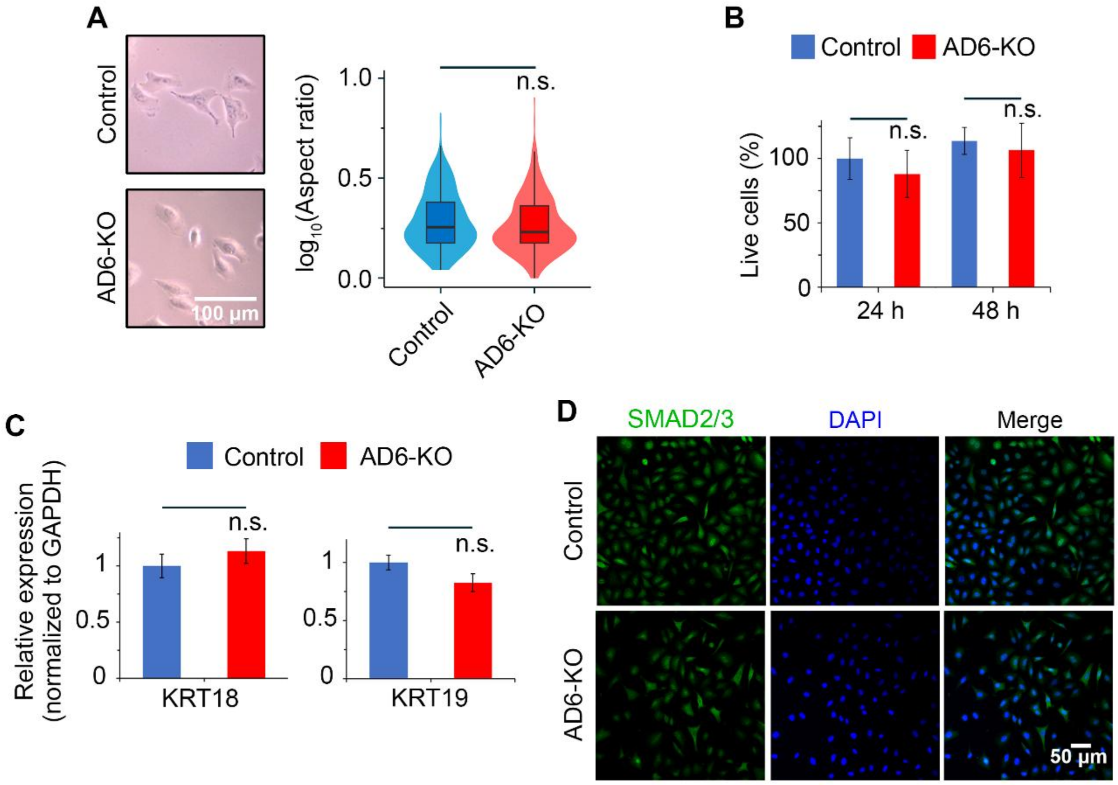

Figure S3. Effect of *ADAMTS6* knockout on EMT-associated cellular functions. Control, parental A549 cells; AD6-KO, A549 cells with *ADAMTS6* gene knockout. (**A**) Cell mor-

phology. Quantification of cell shape was performed by calculating aspect ratio (the ratio of the long axis of the cell to its short axis). (**B**) Cell viability after 24 and 48 h of incubation, evaluated by MTT assay. (**C**) Relative expression of the cadherins *KRT18* and *KRT19*, assessed using RT-qPCR and normalized to *GAPDH* expression. (**D**) IF staining of SMAD2/3 (green) and DAPI staining of nuclei (blue). All experiments were conducted with at least three biological replicates. Data are presented as mean ± SD. N.s., not statistically significant (two-tailed Student's t-test).

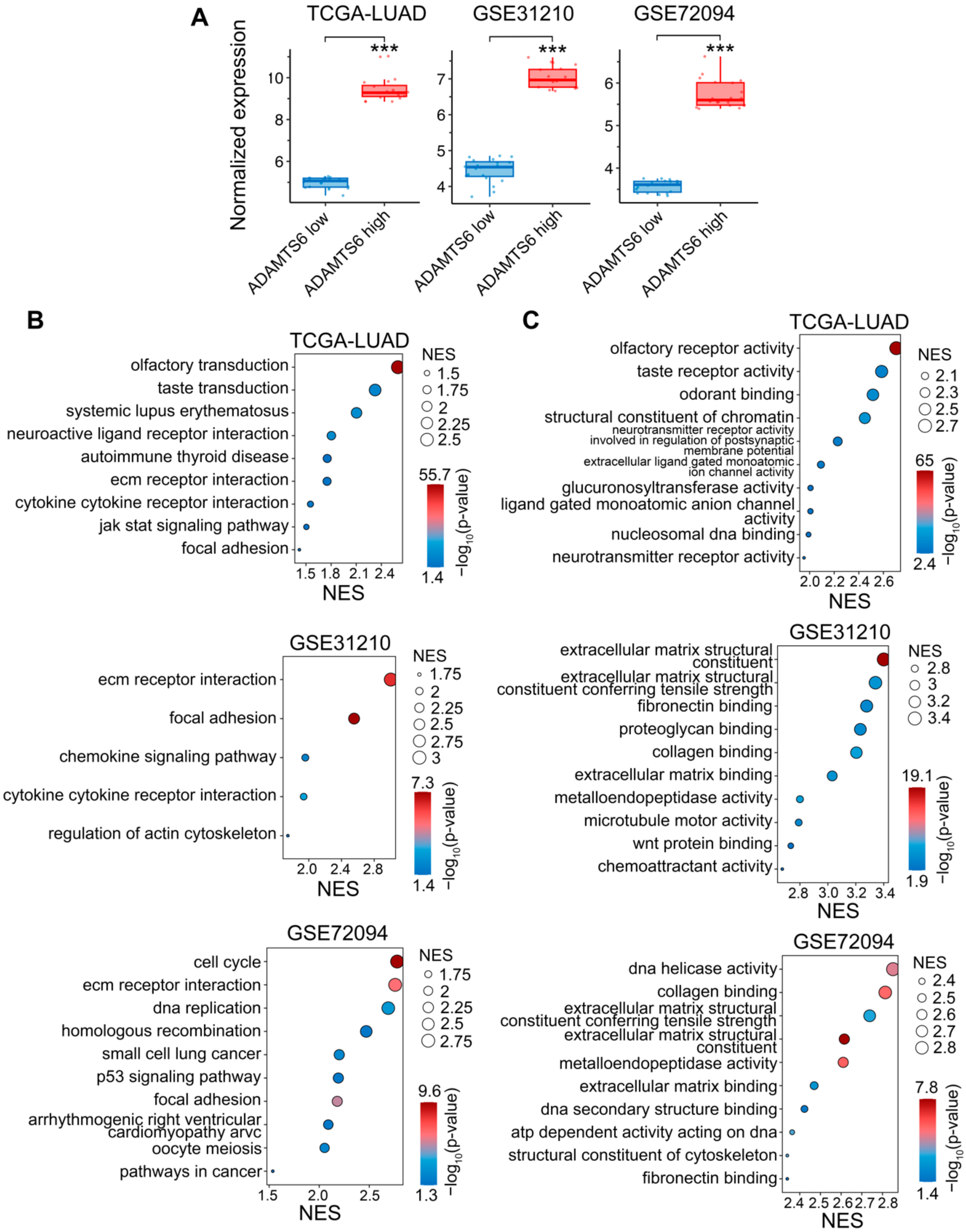

Figure S4. Evaluation of molecular pathways activated in tumors with high *ADAMTS6* expression compared to those with low *ADAMTS6* expression, performed across three LUAD cohorts (TCGA-LUAD, GSE31210, and GSE72094). (**A**) Box plots illustrating

differences in *ADAMTS6* expression between tumors ranked in the top 20 and bottom 20 by *ADAMTS6* expression. *** indicates p-value < 0.001, as calculated by a two-tailed Student's t-test. (**B, C**) Top 10 KEGG (**B**) and top 10 GO-MF (**C**) terms enriched in tumors with high *ADAMTS6* expression, as determined by GSEA.

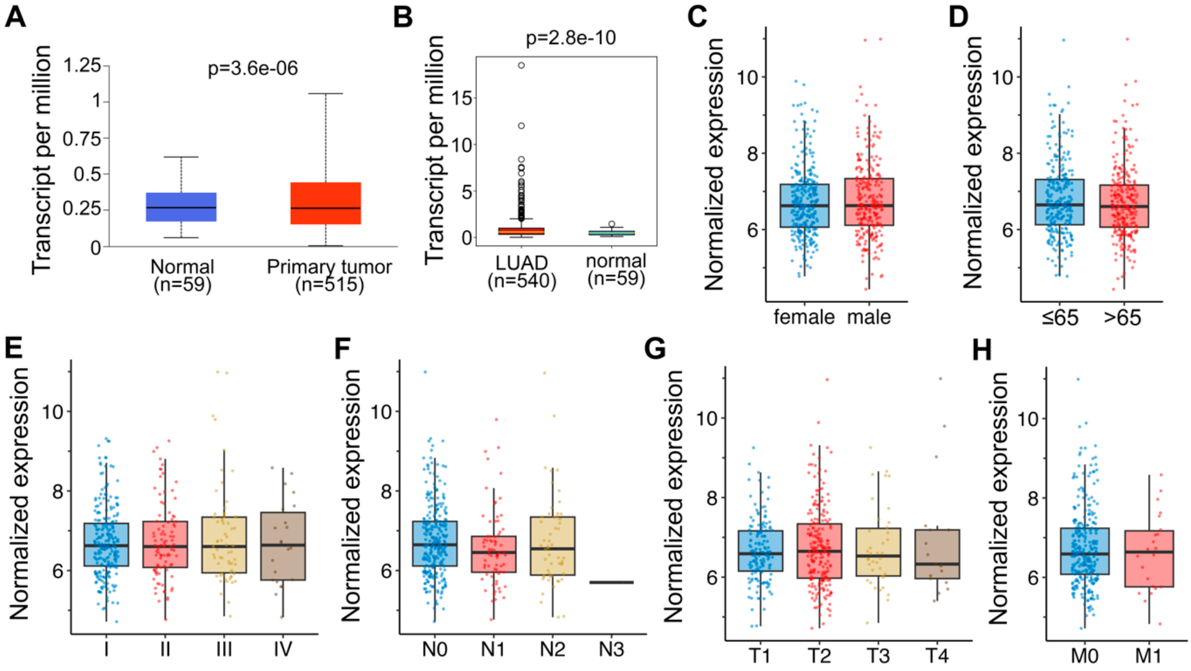

Figure S5. The association between *ADAMTS6* expression and clinicopathological characteristics in TCGA-LUAD cohort. (**A**, **B**) Differences in *ADAMTS6* expression between normal lung tissues and LUAD tumors, as quantified by Students t-test using the UALCAN (**A**) and OncDB (**B**) platforms. Expression of *ADAMTS6* in TCGA-LUAD tumors stratified by gender (**C**), age (**D**), pathological stage (**E**), N stage (**F**), T stage (**G**), and M stage (**H**).

Table S1. Design of differential expression analysis in verification datasets of EMT in lung adenocarcinoma (LUAD), as well as in lung squamous cell carcinoma (SCC) and small airway epithelial cells.

GEO ID	Comparison groups		Platform	Desciption	Ref.
	Test	Control			
GSE277168	A549WT+TGFb	A549WT	Agilent-026652	A549 cells were compared with and without TGF-β1 treatment (5 ng/mL, 48 h).	[1]
GSE277168	A549Lsub+TGFb	A549WT+TGFb	Agilent-026652	A549 cells harbouring CRISP/Cas9-induced mutations impairing SMAD2 phosphorylation (A549L^{sub}) were compared to parental A549 cells under TGF-β1 induction (5 ng/mL, 48 h).	[1]
GSE197132	A549 treated with TGF-β1	A549 control	Illumina HiSeq 3000	A549 cells were compared with and without TGF-β1 treatment (10 ng/mL, 48 h).	[2]
GSE197132	A549 treated with TGF-β1 plus acetate	A549 treated with TGF-β1	Illumina HiSeq 3000	A549 cells induced with TGF-β1 treatment (10 ng/mL, 48 h) were compared with and without sodium acetate (40 mM).	[2]
GSE135402	TD	A549	Illumina NextSeq 500	TD cells were dervied from A549 cells by chronic TGF-β1 treatment (2 ng/mL, 60 d) and compared with parental A549 cells.	[3,4]
GSE95536	TGFb 8h	Control 8h	Illumina HiSeq 4000	SK-MES-1 SCC cells were compared with and without TGF-β1 treatment (2 ng/mL, 8 h).	[5]
GSE95536	TGFb 24h	Control 24h	Illumina HiSeq 4000	SK-MES-1 SCC cells were compared with and without TGF-β1 treatment (2 ng/mL, 24 h).	[5]
GSE95536	TGFb 48h	Control 48h	Illumina HiSeq 4000	SK-MES-1 were compared with and without TGF-β1 treatment (2 ng/mL, 48 h).	[5]
GSE61220	Mes_Control	Epi_Control	Illumina HiSeq 1000	Human small airway epithelial cells were compared with and without chronic TGF-β1 treatment (10 ng/mL, 15 d).	[6]
GSE98979	H1975_TGFb_24h	H1975_Control_24h	Affymetrix Human Gene 2.0 ST Array	H1975 cells were compared with and without TGF-β1 treatment (2 ng/mL, 24 h).	[7]

GEO ID	Comparison groups		Platform	Desciption	Ref.
GSE98979	H1975_TGFb_48h	H1975_Control_48h	Affymetrix Human Gene 2.0 ST Array	H1975 cells were compared with and without TGF-β1 treatment (2 ng/mL, 48 h).	[7]
GSE142620	A549_IL1b_15d	A549_Ctrl_15d	Illumina HiSeq 3000	A549 cells were compared with and without IL-1β treatment (1 ng/mL, 15 d).	[8]
GSE142620	A549_IL1b_21d	A549_Ctrl_21d	Illumina HiSeq 3000	A549 cells were compared with and without IL-1β treatment (1 ng/mL, 21 d).	[8]
GSE32254	A549, +TNFα, control siRNA	A549, -TNFα, control siRNA	Affymetrix Human Gene 1.0 ST Array	A549 cells transfected with control siRNA were compared with and without TNF-α treatment (20 ng/mL, 24 h).	[9]
GSE79688	HCC827_Gef	HCC827_DMSO	Illumina Genome Analyzer IIx	Gefitinib-resistant HCC827 cells cultured in 3 μM gefitinib were compared to control cells cultured in DMSO.	[10]
GSE79688	HCC4006_Gef	HCC4006_DMSO	Illumina Genome Analyzer IIx	Gefitinib-resistant HCC4006 cells cultured in 3 μM gefitinib were compared to control cells cultured in DMSO.	[10]
GSE269441	0628-EGF	0628-Control	Illumina NovaSeq 6000	H1975 cells were compared with and without EGF treatment (concentration and time point unspecified).	[11]
GSE269441	0628-siEGFR	0628-NC	Illumina NovaSeq 6000	H1975 cells targeted with EGFR siRNA were compared to cells targeted with a negative control siRNA (siNC).	[11]
GSE115456	H1975 PMA	H1975 control	Affymetrix Human Genome U133 Plus 2.0 Array	H1975 cells were compared with and without PMA treatment (100 nM, 24 h).	[12]
GSE62359	H1993 BRMS1 KD/Twist1 KD	H1993 BRMS1 KD	Illumina HumanHT-12 V4.0 expression beadchip	EMT was induced in H1993 cells by knocking down BRMS1 with shRNA. These BRMS1-deficient cells were then compared with and without Twist1 knockdown using shRNA.	[13]
GSE131594	HCC827 Dormant	HCC827 DMSO	Illumina NextSeq 500	HCC827 cells were treated with 100 nM osimertinib and 30 nM trametinib for 2 wk and compared to DMSO-treated control cells.	[14]

GEO ID	Comparison groups		Platform	Desciption	Ref.
GSE131594	PC9 Dormant	PC9 DMSO	Illumina NextSeq 500	PC9 cells were treated with 100 nM osimertinib and 30 nM trametinib for 2 wk and compared to DMSO-treated control cells.	[14]
GSE131601	HCC4006 YAP1 KO DMSO	HCC4006 CTRL DMSO	Illumina NextSeq 500	HCC4006 cells with and without CRISPR/Cas9-induced YAP1 knock-out were compared after 24 h of DMSO treatment.	[14]
GSE131601	HCC4006 YAP1 KO OT	HCC4006 YAP1 KO DMSO	Illumina NextSeq 500	HCC4006 cells with YAP1 knockout treated with 100 nM osimertinib and 30 nM trametinib for 24 h were compared to YAP1 knockout cells treated with DMSO.	[14]
GSE232714	siRAVER1	siC control knock-down	Illumina NovaSeq 6000	A549 cells targeted with RAVER1 siRNA were compared to cells targeted with a control siRNA (siC).	[15]

The names of the comparison groups correspond to those used in the original GEO entries. References (ref.) to the studies in which the cell models were used and RNA-seq data were obtained:

1. Nitschkowski, D.; Vierbuchen, T.; Heine, H.; Behrends, J.; Reiling, N.; Reck, M.; Rabe, K.F.; Kugler, C.; Ammerpohl, O.; Drömann, D.; et al. SMAD2 Linker Phosphorylation Impacts Overall Survival, Proliferation, TGFβ1-Dependent Gene Expression and Pluripotency-Related Proteins in NSCLC. *British Journal of Cancer* **2025**, *133*, 52–65, doi:10.1038/s41416-025-02970-1.
2. Lyu, J.; Pirooznia, M.; Li, Y.; Xiong, J. The Short-Chain Fatty Acid Acetate Modulates Epithelial-to-Mesenchymal Transition. *Molecular Biology of the Cell* **2022**, *33*, br13, doi:10.1091/mbc.E22-02-0066.
3. Kwon, O.-S.; Kwon, E.-J.; Kong, H.-J.; Choi, J.-Y.; Kim, Y.-J.; Lee, E.-W.; Kim, W.; Lee, H.; Cha, H.-J. Systematic Identification of a Nuclear Receptor-Enriched Predictive Signature for Erastin-Induced Ferroptosis. *Redox Biology* **2020**, *37*, 101719, doi:10.1016/j.redox.2020.101719.
4. Bae, G.Y.; Hong, S.K.; Park, J.R.; Kwon, O.S.; Kim, K.T.; Koo, J.H.; Oh, E.; Cha, H.J. Chronic TGFβ Stimulation Promotes the Metastatic Potential of Lung Cancer Cells by Snail Protein Stabilization through Integrin B3-Akt-GSK3β Signaling. *Oncotarget* **2016**, *7*, 25366–25376, doi:10.18632/oncotarget.8295.
5. Dvornikov, D.; Schneider, M.A.; Ohse, S.; Szczygieł, M.; Titkova, I.; Rosenblatt, M.; Muley, T.; Warth, A.; Herth, F.J.; Dienemann, H.; et al. Expression Ratio of the TGFβ-Inducible Gene MYO10 Is Prognostic for Overall Survival of Squamous Cell Lung Cancer Patients and Predicts Chemotherapy Response. *Scientific Reports* **2018**, *8*, 9517, doi:10.1038/s41598-018-27912-1.
6. Tian, B.; Li, X.; Kalita, M.; Widen, S.G.; Yang, J.; Bhavnani, S.K.; Dang, B.; Kudlicki, A.; Sinha, M.; Kong, F.; et al. Analysis of the TGFβ-Induced Program in Primary Airway Epithelial Cells Shows Essential Role of NF-κB/RelA Signaling Network in Type II Epithelial Mesenchymal Transition. *BMC Genomics* **2015**, *16*, 529, doi:10.1186/s12864-015-1707-x.
7. Gladilin, E.; Ohse, S.; Boerries, M.; Busch, H.; Xu, C.; Schneider, M.; Meister, M.; Eils, R. TGFβ-Induced Cytoskeletal Remodeling Mediates Elevation of Cell Stiffness and Invasiveness in NSCLC. *Scientific Reports* **2019**, *9*, 7667, doi:10.1038/s41598-019-43409-x.
8. Li, R.; Ong, S.L.; Tran, L.M.; Jing, Z.; Liu, B.; Park, S.J.; Huang, Z.L.; Walser, T.C.; Heinrich, E.L.; Lee, G.; et al. Chronic IL-1β-Induced Inflammation Regulates Epithelial-to-Mesenchymal Transition Memory Phenotypes via Epigenetic Modifications in Non-Small Cell Lung Cancer. *Scientific Reports* **2020**, *10*, 377, doi:10.1038/s41598-019-57285-y.
9. Shiozaki, A.; Bai, X.; Shen-Tu, G.; Moodley, S.; Takeshita, H.; Fung, S.-Y.; Wang, Y.; Keshavjee, S.; Liu, M. Claudin 1 Mediates TNFα-Induced Gene Expression and Cell Migration in Human Lung Carcinoma Cells. *PLoS One* **2012**, *7*, e38049, doi:10.1371/journal.pone.0038049.
10. Ware, K.E.; Hinz, T.K.; Kleczko, E.; Singleton, K.R.; Marek, L.A.; Helfrich, B.A.; Cummings, C.T.; Graham, D.K.; Astling, D.; Tan, A.-C.; et al. A Mechanism of Resistance to Gefitinib Mediated by Cellular Repro-

gramming and the Acquisition of an FGF2-FGFR1 Autocrine Growth Loop. *Oncogenesis* **2013**, *2*, e39–e39, doi:10.1038/oncsis.2013.4.
11. Ao, L.; Jia, W.; Gong, Q.; Cui, J.; Wang, J.; Yu, Y.; Fu, C.; Li, H.; Wei, J.; Wang, R.; et al. Targeting GFPT2 to Reinvigorate Immunotherapy in EGFR-Mutated NSCLC. *bioRxiv* **2024**, 2024.03.01.582888, doi:10.1101/2024.03.01.582888.
12. Weber, R.; Meister, M.; Muley, T.; Thomas, M.; Sültmann, H.; Warth, A.; Winter, H.; Herth, F.J.F.; Schneider, M.A. Pathways Regulating the Expression of the Immunomodulatory Protein Glycodelin in Non-Small Cell Lung Cancer. *International Journal of Oncology* **2019**, *54*, 515–526, doi:10.3892/ijo.2018.4654.
13. Liu, Y.; Mayo, M.W.; Xiao, A.; Hall, E.H.; Amin, E.B.; Kadota, K.; Adusumilli, P.S.; Jones, D.R. Loss of BRMS1 Promotes a Mesenchymal Phenotype through NF-κB-Dependent Regulation of Twist1. *Molecular and Cellular Biology* **2015**, *35*, 303–317, doi:10.1128/MCB.00869-14.
14. Kurppa, K.J.; Liu, Y.; To, C.; Zhang, T.; Fan, M.; Vajdi, A.; Knelson, E.H.; Xie, Y.; Lim, K.; Cejas, P.; et al. Treatment-Induced Tumor Dormancy through YAP-Mediated Transcriptional Reprogramming of the Apoptotic Pathway. *Cancer Cell* **2020**, *37*, 104-122.e12, doi:10.1016/j.ccell.2019.12.006.
15. Wedler, A.; Bley, N.; Glaß, M.; Müller, S.; Rausch, A.; Lederer, M.; Urbainski, J.; Schian, L.; Obika, K.-B.; Simon, T.; et al. RAVER1 Hinders Lethal EMT and Modulates miR/RISC Activity by the Control of Alternative Splicing. *Nucleic Acids Research* **2024**, *52*, 3971–3988, doi:10.1093/nar/gkae046.

Table S2. Protospacer and PCR primer sequences.

Name	Description	5′-3′ sequence
AD6_p4-1_t	Protospacer “4-1” top strand	CACCGGAGTTCTCTTCGTTCAGTG
AD6_p4-1_b	Protospacer “4-1” bottom strand	AAACCACTGAACGAAGAGAACTCC
AD6_pe-b-t	Protospacer “e-b” top strand	CACCGCTGTATGATCACTCTCATTG
AD6_pe-b-b	Protospacer “e-b” bottom strand	AAACCAATGAGAGTGATCATACAGC
AD6_4F	Forward primer 4F	TCTGTGTCTACATCAGACATCTT
AD6_4R	Reverse primer 4R	TTCCCCCAACCACTATGAAC
AD6_5F	Forward primer 5F	GAGTCGTTGTGAGATTGGAGG
AD6_5R	Reverse primer 5R	TGTAGTCAAACTCCTACTTCCTT

Table S3. RT-qPCR primer sequences.

Gene	Description	Forward primer	Reverse primer
GAPDH	Housekeeping	GTGAAGGTCGGAGTCAAC	TGGAATTTGCCATGGGTG
ADAMTS6	Gene under study	AAACGGTATCGCTCCTGTAAC	GTTTTACCCCACCTCCAGTATAG
CDH1	Epithelial marker	CCAGAATAAAGACCAAGTGAC	CCAAGAATCCCCAGAATGGCAGGAAT
KRT18	Epithelial marker	TCAAGGTCAAGCTGGAG	CTTTGGTGTCATTGGTCTC
KRT19	Epithelial marker	AGGGTCTTGAGATTGAGC	TGGTACTCCTGATTCTGC
CDH2	Mesenchymal marker	GCTACTTTCCTTGCTTCTG	GAGGTAACACTTGAGGG
VIM	Mesenchymal marker	TGAGATTGCCACCTACAG	TGAGAAGTTTCGTTGATAACC
TWIST1	Mesenchymal marker	GTACATCGACTTCCTCTAC	CAATGACATCTAGGTCTCC

Supplementary Results

Sanger sequencing of *ADAMTS6* alleles was performed on wild type A549 cells and CRISP/Cas9-generated 202 clones using 4F/4R primers. The resulting sequences are shown below, with text color-coordinated to match Fig. 4C:

Wild type:
TCTGTGTCTACATCAGACATCTTTCTAAGAGGTCACACAGGGACCCAGGTTCCTTCTTTTT-
GGTTTCTACCTACCCTAGGTATTTGGAGTTCTCTTCGTTCAGTGGGGAAAGAGAGTAGAA-
GATTCCACTGCAGAGGTATTCAGAGGTT-
GGGCTGAAAGTGTTACATAACCCTCTGCCCATATTTTTATTGTGCAAAACTCAATCACAAA-
GCTATATCTAAACACAGAGGACATAGTTATGTGCATAATGTGGAACTGAAATAAGTTTTAG-
TGTTCACATAAAATCTGTTACCTCACAGAGTCGTTGTGAGATTGGAGGAAATCAC-
GTATGTGACATGCTTAGTACAGTGCATAGCATATGGAGGTATTTAAATGTCCTTTTTAAA-
GAATAGGAACATTTATATAATGTTTTAATTGAGTACAGA-
TAATTCGCTAATTTTTTTATTAACGTGTTTTAACCCTGTAAATGGA-
GAAGTGTCCATTTACTTTTAGCTCTTGGTAATAATGATAACTAATATTTAAAATGAT-
TCTCTTTGGTAAGTTCTTTGTTTATTTTTCTTTTAGCATGGTGTTATTGCTACAGAAGATGAA-
GAGTATTTTATCGAACCTTTAAAGAATACCACAGAGGATTCCAAGCATTTTAG-
TTATGAAAATGGCCACCCTCATGTTATTTACAAAAAGTCTGCCCTTCAACAACGACATCTG-
TATGATCACTCTCATTGTGGGGTTTCGGGTGAGTGAGTGTGCAGCTTTTACAAAGCG-
TATTGTACTGGATTGCTGCTTTTTCTTTCTTCTGGCAAGTATAGTAAGGTTAGCAA-
TAAAAGGAAGTAGGAGTTTGACTACAATTATAATGTCCAGAGCCCTTTTAATAGATGTCAC-
CTACCAGTAGTTTTAAATCAATTTGGGAGCTCTTTGAGAAGTTTTTAAGCTGTAAGTTT-
GTATAAAATTCATTTTCTTTTTTTGGTTGAAGGGAAAAAAATAAC-
CATTTTATTTTACTTAGGTAATAGCTATAGCTTTTTTGCACAGTTATACTGTAG-
TACTCTGTGGTGTTCGTTTAGCCTGATATTTTTATGCTAGTAGCAGGAATCTAGACACTTGAA-
TAAGACTTGAAGAGTCTTAAGATAGTATTCAAAAGTAGTGTTAGCACTGAGCGAGGA-
TACTATAGAAATATGTAGTACGGGCAACAAACTTAGACTTCAGTGGTCAAGGAAA-
GCTTCATGGAAGAAGTGACTTCTAAGCTGAGGGCTGGGGGAAAAGTGAAGGTAGCTGTG-
TAGAAGTGGAGAAAGCAGTGGCTATTAGTGTTTCACATAGAGGAAATAGTGTTTATGAA-
GCTCCTAAACCAAAAGGGAGAATGTCTTATAATTGTTTTATAAGACATGTTTTATAAATT-
GACAGACAGTTCATGTAACTGGATATTAGAATGTGGGCTGGAGGGA-
TAAAGGTGAGGTTCATAGTGGTTGGGGGAA

4-1+e-b clone 202/allele 1 (1047 bp deletion highlighted in orange):
TCTGTGTCTACATCAGACATCTTTCTAAGAGGTCACACAGGGACCCAGGTTCCTTCTTTTT-
GGTTTCTACCTACCCTAGGTATTTGGAGTTCTCTTCGTTCAGTGGGGAAAGAGAGTAGAA-
GATTCCACTGCAGAGGTATTCAGAGGTT-
GGGCTGAAAGTGTTACATAACCCTCTGCCCATATTTTTATTGTGCAAAACTCAATCACAAA-
GCTATATCTAAACACAGAGGACATAGTTATGTGCATAATGTGGAACTGAAATAAGTTTTAG-
TGTTCACATAAAATCTGTTACCTCACAGAGTCGTTGTGAGATTGGAGGAAATCAC-
GTATGTGACATGCTTAGTACAGTGCATAGCATATGGAGGTATTTAAATGTCCTTTTTAAA-
GAATAGGAACATTTATATAATGTTTTAATTGAGTACAGA-
TAATTCGCTAATTTTTTTATTAACGTGTTTTAACCCTGTAAATGGA-
GAAGTGTCCATTTACTTTTAGCTCTTGGTAATAATGATAACTAATATTTAAAATGAT-
TCTCTTTGGTAAGTTCTTTGTTTATTTTTCTTTTAGCATGGTGTTATTGCTACAGAAGATGAA-
GAGTATTTTATCGAACCTTTAAAGAATACCACAGAGGATTCCAAGCATTTTAG-
TTATGAAAATGGCCACCCTCATGTTATTTACAAAAAGTCTGCCCTTCAACAACGACATCTG-
TATGATCACTCTCATTGTGGGGTTTCGGGTGAGTGAGTGTGCAGCTTTTACAAAGCG-
TATTGTACTGGATTGCTGCTTTTTCTTTCTTCTGGCAAGTATAGTAAGGTTAGCAA-
TAAAAGGAAGTAGGAGTTTGACTACAATTATAATGTCCAGAGCCCTTTTAATAGATGTCAC-
CTACCAGTAGTTTTAAATCAATTTGGGAGCTCTTTGAGAAGTTTTTAAGCTGTAAGTTT-
GTATAAAATTCATTTTCTTTTTTTGGTTGAAGGGAAAAAAATAAC-
CATTTTATTTTACTTAGGTAATAGCTATAGCTTTTTTGCACAGTTATACTGTAG-
TACTCTGTGGTGTTCGTTTAGCCTGATATTTTTATGCTAGTAGCAGGAATCTAGACACTTGAA-
TAAGACTTGAAGAGTCTTAAGATAGTATTCAAAAGTAGTGTTAGCACTGAGCGAGGA-
TACTATAGAAATATGTAGTACGGGCAACAAACTTAGACTTCAGTGGTCAAGGAAA-

GCTTCATGGAAGAAGTGACTTCTAAGCTGAGGGCTGGGGGAAAAGTGAAGGTAGCTGTG-
TAGAAGTGGAGAAAGCAGTGGCTATTAGTGTTTCACATAGAGGAAATAGTGTTTATGAA-
GCTCCTAAACCAAAAGGGAGAATGTCTTATAATTGTTTTATAAGACATGTTTTATAAATT-
GACAGACAGTTCATGTAACTGGATATTAGAATGTGGGCTGGAGGGA-
TAAAGGTGAGGTTCATAGTGGTTGGGGGAA

4-1+e-b clone 202/allele 2 (618 bp inversion colored in blue, insertions highlighted in green):

TCTGTGTCTACATCAGACATCTTTCTAAGAGGTCACACAGGGACCCAGGTTCCTTCTTTTTT-
GGTTTCTACCTACCCTAGGTATTTGGAGTTCTCTTCGTTCATGAGAGTGATCATA-
CAGATGTCGTTGTTGAAGGGCAGACTTTTTGTAAA-
TAACATGAGGGTGGCCATTTTCATAACTAAAATGCTTGGAATCCTCTGTGG-
TATTCTTTAAAGGTTCGATAAAATACTCTTCATCTTCTGTAGCAATAACACCATGCTAAAA-
GAAAAATAAACAAAGAACTTACCAAAGAGAATCATTTTAAATATTAGTTATCATTATTAC-
CAAGAGCTAAAAGTAAATGGACACTTCTCCATTTACAGGGTTAAAACACGTTAA-
TAAAAAAATTAGCGAATTATCTGTACTCAATTAAAACATTATATAAATGTTCC-
TATTCTTTAAAAAGGACATTTAAATACCTCCATATGCTATGCACTGTACTAA-
GCATGTCACATACGTGATTTCCTCCAATCTCACAACGACTCTGTGAGGTAACAGAT-
TTTATGTGAACACTAAAACTTATTTCAGTTCCACATTATGCACATAACTATGTCCTCTGTGTT-
TAGATATAGCTTTGTGATTGAGTTTTGCACAATAAAAATATGGGCAGAGGGTTATGTAACAC-
TTTCAGCCCAACCTCTGAATACCTCTGCAGTGGAATCTTCTACTCTCTTTCCCCACTTT-
GTGGGGTTTCGGGTGAGTGAGTGTGCAGCTTTTACAAAGCGTATTGTACTG-
GATTGCTGCTTTTTCTTTCTTCTGGCAAGTATAGTAAGGTTAGCAATAAAAGGAAGTAGGAG-
TTTGACTACAATTATAATGTCCAGAGCCCTTTTAATAGATGTCACCTACCAGTAG-
TTTTAAATCAATTTGGGAGCTCTTTGAGAAGTTTTTAAGCTGTAAGTTT-
GTATAAAATTCATTTTCTTTTTTTGGTTGAAGGGAAAAAAATAAC-
CATTTTATTTTACTTAGGTAATAGCTATAGCTTTTTTGCACAGTTATACTGTAG-
TACTCTGTGGTGTTCGTTTAGCCTGATATTTTTATGCTAGTAGCAGGAATCTAGACACTTGAA-
TAAGACTTGAAGAGTCTTAAGATAGTATTCAAAAGTAGTGTTAGCACTGAGCGAGGA-
TACTATAGAAATATGTAGTACGGGCAACAAACTTAGACTTCAGTGGTCAAGGAAA-
GCTTCATGGAAGAAGTGACTTCTAAGCTGAGGGCTGGGGGAAAAGTGAAGGTAGCTGTG-
TAGAAGTGGAGAAAGCAGTGGCTATTAGTGTTTCACATAGAGGAAATAGTGTTTATGAA-
GCTCCTAAACCAAAAGGGAGAATGTCTTATAATTGTTTTATAAGACATGTTTTATAAATT-
GACAGACAGTTCATGTAACTGGATATTAGAATGTGGGCTGGAGGGA-
TAAAGGTGAGGTTCATAGTGGTTGGGGGAA

CRISP/Cas9 generated a deletion in allele 1 and an inversion in allele 2, as shown by the sequences. Interestingly, DNA repair appears to have introduced insertions in allele 2: deoxyadenosine (A) at the beginning and a deoxythymidine (T) at the end of the inversion.
